# Crystal structure of di­chlorido­bis­[2-(phenyl­diazen­yl)pyridine-κ*N* ^1^]zinc

**DOI:** 10.1107/S2056989015019143

**Published:** 2015-10-24

**Authors:** Luksamee Vittaya, Nararak Leesakul, Saowanit Saithong, Kittipong Chainok

**Affiliations:** a Faculty of Science and Fisheries Technology, Rajamangala University of Technology Srivijaya, Sikao, Trang 92150, Thailand; b Department of Chemistry and Center for Innovation in Chemistry, Faculty of Science, Prince of Songkla University, Hat Yai, Songkhla 90112, Thailand; c Department of Physics, Faculty of Science and Technology, Thammasat University, Khlong Luang, Pathum Thani 12121, Thailand

**Keywords:** crystal structure, zinc complex, C—H⋯Cl inter­actions

## Abstract

The structure of the title complex, [ZnCl_2_(C_11_H_9_N_3_)_2_], comprises two 2-(phenyl­diazen­yl)pyridine ligands coordin­ating to a central Zn^II^ dichloride unit *via* the pyridyl N-atom donors, resulting in a slightly distorted tetra­hedral geometry. The complex exhibits twofold rotation symmetry, with the rotation axis bis­ecting the zinc cation. The structure is stabilized by weak inter­molecular C—H⋯Cl inter­actions [C⋯Cl = 3.411 (2) and 3.675 (2) Å], connecting neighbouring mol­ecules into layers perpendicular to the *c* axis.

## Related literature   

For background to diazenyl­pyridine compounds, see: Krause & Krause (1980 ▸). For applications of diazenyl­pyridine complexes, see: Wong & Giandomenico (1999 ▸); Wu *et al.* (2006 ▸); Hotze *et al.* (2004 ▸); Velders *et al.* (2000 ▸); Barf & Sheldon (1995 ▸). For applications of zinc–diazenyl complexes, see: Saha *et al.* (2014 ▸); Dutta *et al.* (2014 ▸); Datta *et al.* (2014 ▸); Zhang *et al.* (2012 ▸). For related structures, see: Leesakul *et al.* (2011 ▸); Panneerselvam *et al.* (2000 ▸); Steffen & Palenik (1976 ▸).
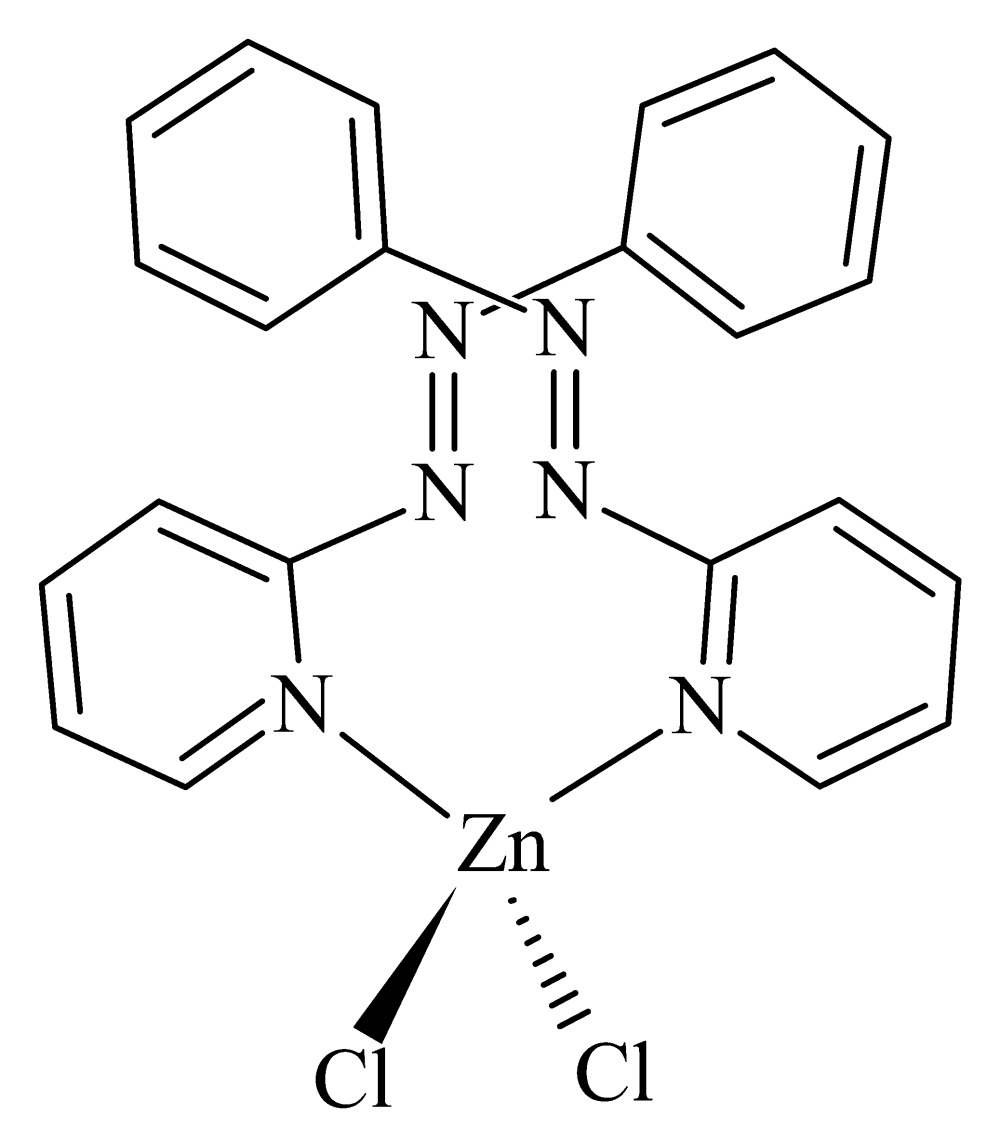


## Experimental   

### Crystal data  

[ZnCl_2_(C_11_H_9_N_3_)_2_]
*M* *_r_* = 502.69
Orthorhombic, 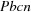

*a* = 13.7960 (4) Å
*b* = 10.1905 (3) Å
*c* = 16.1305 (5) Å
*V* = 2267.76 (12) Å^3^
*Z* = 4
Mo *K*α radiation
μ = 1.34 mm^−1^
*T* = 298 K
0.36 × 0.32 × 0.30 mm

### Data collection  

Bruker APEXII CCD diffractometer
Absorption correction: multi-scan (*SADABS*; Bruker, 2013 ▸) *T*_min_ = 0.708, *T*_max_ = 0.746
65168 measured reflections
2820 independent reflections
2160 reflections with *I* > 2σ(*I*)
*R*_int_ = 0.041

### Refinement  

*R*[*F* ^2^ > 2σ(*F* ^2^)] = 0.030
*wR*(*F* ^2^) = 0.083
*S* = 1.06
2820 reflections
141 parameters
H-atom parameters constrained
Δρ_max_ = 0.35 e Å^−3^
Δρ_min_ = −0.22 e Å^−3^

### 

Data collection: *SMART* (Bruker, 2013 ▸); cell refinement: *SAINT* (Bruker, 2013 ▸); data reduction: *SAINT*; program(s) used to solve structure: *SHELXS97* (Sheldrick, 2008 ▸); program(s) used to refine structure: *SHELXL2014* (Sheldrick, 2015 ▸); molecular graphics: *Mercury* (Macrae *et al.*, 2008 ▸); software used to prepare material for publication: *WinGX* (Farrugia, 2012 ▸) and *publCIF* (Westrip, 2010 ▸).

## Supplementary Material

Crystal structure: contains datablock(s) I, New_Global_Publ_Block. DOI: 10.1107/S2056989015019143/zl2646sup1.cif

Structure factors: contains datablock(s) I. DOI: 10.1107/S2056989015019143/zl2646Isup2.hkl

**Fig. 1.**
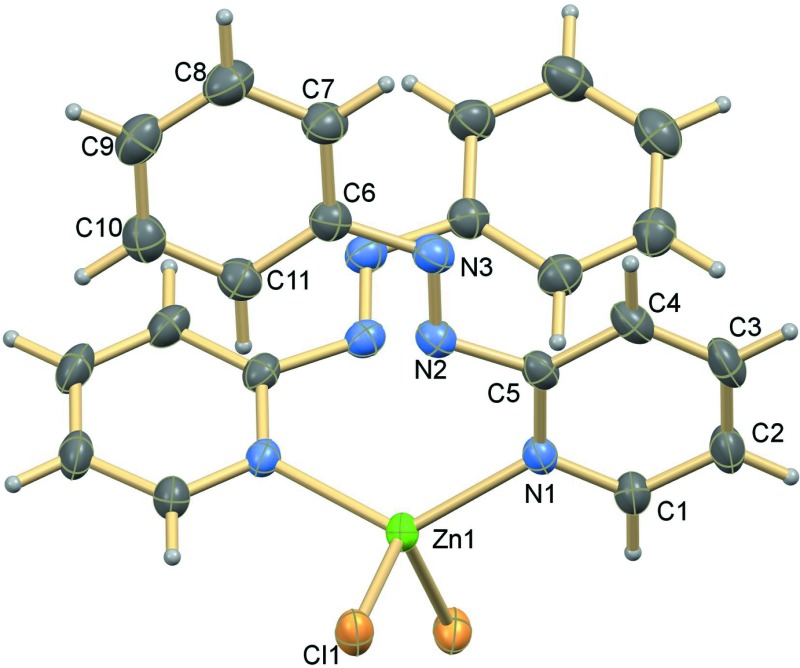
Molecular structure of [Zn(C11H9N3)2Cl2] with thermal ellipsoids plotted at the 30% probability level. Non-labelled atoms are created by the twofold symmetry axis [symmetry operator: (i) -x + 2, y, -z + 3/2].

11 9 3 2 2 x y -z . DOI: 10.1107/S2056989015019143/zl2646fig1.tif
Mol­ecular structure of [Zn(C_11_H_9_N_3_)_2_Cl_2_] with thermal ellipsoids plotted at the 30% probability level. Non-labelled atoms are created by the twofold symmetry axis [symmetry operator: (i) −*x* + 2, *y*, *-z* + 3/2].

**Fig. 2.**
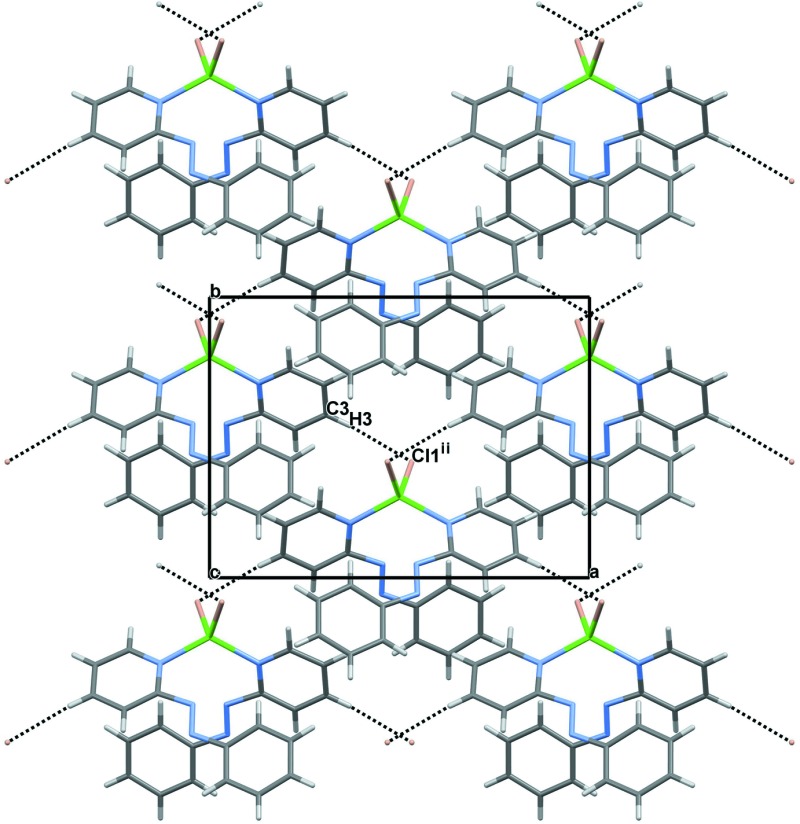
Two–dimensional interaction sheet of [Zn(C11H9N3)2Cl2] plotted down c, formed through weak C–H···Cl interactions.

11 9 3 2 2 c . DOI: 10.1107/S2056989015019143/zl2646fig2.tif
Two–dimensional inter­action sheet of [Zn(C_11_H_9_N_3_)_2_Cl_2_] plotted down *c*, formed through weak C–H⋯Cl inter­actions.

**Fig. 3.**
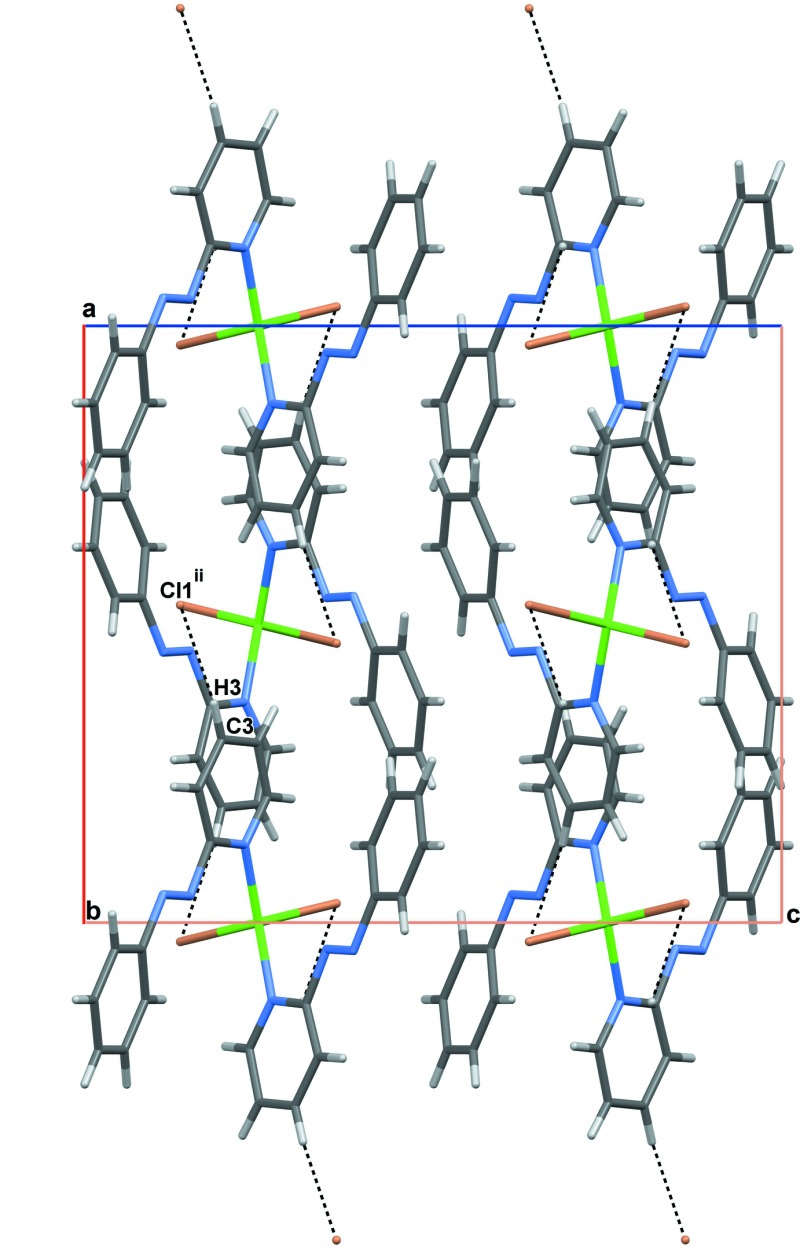
Two–dimensional interaction sheet of [Zn(C11H9N3)2Cl2] plotted down b axis, formed through weak C–H···Cl interactions.

**Fig. 4.**
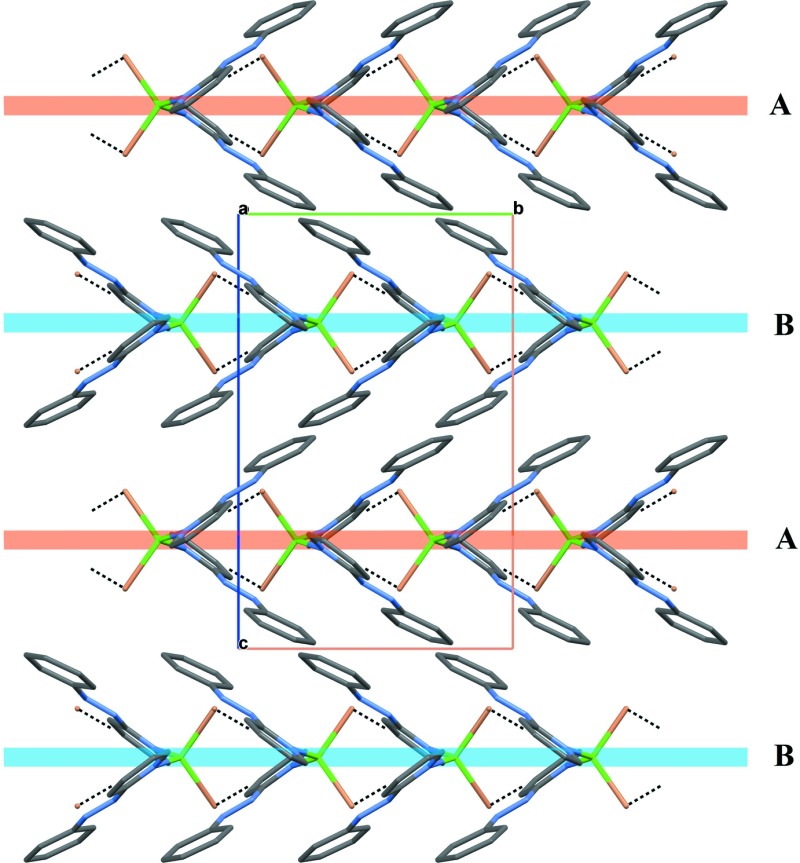
The arrangement of two-dimensional layers plotted down the a axis showing a lateral view of alternating C···Cl contact directions of adjacent sheets (A and B layers). H atoms are omitted for clarity.

11 9 3 2 2 b . DOI: 10.1107/S2056989015019143/zl2646fig3.tif
Two–dimensional inter­action sheet of [Zn(C_11_H_9_N_3_)_2_Cl_2_] plotted down *b* axis, formed through weak C–H⋯Cl inter­actions.

a . DOI: 10.1107/S2056989015019143/zl2646fig4.tif
The arrangement of two-dimensional layers plotted down the *a* axis showing a lateral view of alternating C⋯Cl contact directions of adjacent sheets (A and B layers). H atoms are omitted for clarity.

CCDC reference: 1430587

Additional supporting information:  crystallographic information; 3D view; checkCIF report

## Figures and Tables

**Table 1:** Hydrogen-bond geometry (, )

*D*H*A*	*D*H	H*A*	*D* *A*	*D*H*A*
C3H3Cl1^i^	0.93	2.75	3.675(2)	173
C1H1Cl1	0.93	2.81	3.411(2)	124

Symmetry code: (i) 
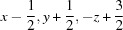
.

## supplementary crystallographic information

### S1. Comment

Nitro­gen containing heteroaromatic systems like pyridines are some of the most investigated organic compounds primarily because of their importance in the pharmaceutical and chemical industries. Pyridine derivatives have been widely studied in analytical chemistry as an acid-base, redox and biomedical agent and for their photo-physical properties. A common synthetic pyridine derivative is 2-(phenyl­diazenyl)pyridine (Krause & Krause, 1980). This compound is a well known ligand with a basic nitro­gen atom on the pyridine ring able to coordinate to transition metal complexes. It has various applications, for example as a chemotherapeutic drug (Wong & Giandomenico, 1999), it has anti­cancer properties (Wu *et al.*, 2006; Hotze *et al.*, 2004; Velder *et al.*, 2000) and is active in catalysing the epoxidation of olefins (Barf & Sheldon 1995). Coordination compounds of Zn^II^ incorporating the diazenyl moiety were shown to have photochromic properties (Saha *et al.*, 2014; Dutta *et al.*, 2014; Datta *et al.*, 2014) as well as non-linear optical properties, which were investigated for the storage of optical information (Zhang *et al.*, 2012).

We here report the preparation and crystal structure of a new Zn^II^ complex with the well known diazenyl­pyridine ligand 2-(phenyl­diazenyl)pyridine (C_11_H_9_N_3_), or azpy. The molecular structure of the title complex, [Zn(C_11_H_9_N_3_)_2_Cl_2_], is slightly distorted tetra­hedral as illustrated in Fig.1. The Zn atom is four coordinated by two azpy ligands *via* two N(pyridine) atoms [Zn1—N1 and Zn1—N1^i^ = 2.0618 (15) Å] and two Cl^-^ ions [Zn1—Cl1 and Zn1—Cl1^i^ = 2.2472 (5) Å ; (i): -x+2,y,-z+3/2]. These values compare well with those of other related di­chloro Zn^II^ compounds with pyridine ligands, such as [Zn(N,N-di­ethyl-4-[(pyridin-2-yl-*k*N)diazenyl]aniline)_2_Cl_2_] (Leesakul *et al.*, 2011) with Zn—N distances of 2.0513 (9) and 2.0439 (19) Å or [Zn(pyridine)_2_Cl_2_] (Steffen & Palenik, 1976) with Zn —N distances of 2.046 (5) and 2.052 (6) Å. The reported Zn—Cl bond distances in the complex of Leesakul *et al.* averaged to 2.264 (6) Å, those in [Zn(pyridine)_2_Cl_2_] (Steffen & Palenik, 1976) averaged to 2.2215 Å, which are thus slightly longer and shorter respectively than those of the title complex [Zn(azpy)_2_Cl_2_].

The azo (N═N) distance in the Zn^II^ complex is 1.244 (2) Å, which is comparable to that in the free azpy ligand, 1.248 (4) Å (Panneerselvam *et al.*, 2000), which is expected as the azo nitro­gen of the azpy ligand is not metal coordinated. All N—Zn—Cl, Cl—Zn—Cl and N—Zn—N bond angles deviate somewhat from the ideal from 109.5°, especially for the angle N1—Zn1—N1^i^ = 124.45 (9)°, probably due to steric demands of the azpy ligand. The torsion angle of the pyridine-azo-phenyl atoms, C5—N2—N3—C6 is 179.59 (15)°. The dihedral angle of the mean planes between the pyridine and the phenyl ring in the ligand molecule is 11.9 (1)°.

### S2. Supra­molecular features

Weak intra and inter­molecular C—H···Cl inter­actions (C···Cl = 3.411 (2) and 3.675 (2) Å; see Table 1, Hydrogen-bond geometry) connect neighboring molecules into layers perpendicular to the *c*-axis (Fig. 2–4).

### S3. Experimental

An aceto­nitrile solution (10 mL) of 2-(phenyl­diazenyl)pyridine (azpy) (0.183 g, 1.0 mmol) was added dropwise to zinc(II) chloride (0.068 g, 0.50 mmol), then refluxed for 4 h. After being filtered, the filtrate was left standing overnight at 4°C. Orange crystals were obtained (yield 71.31%, 0.179 g). Anal. Calcd for ZnC_22_H_18_N_6_Cl_2_: C, 52.56; H, 3.61; N, 16.72. Found: C, 52.56; H, 3.55; N, 16.96.

### S4. Refinement

All H atoms of aromatic carbon were positioned geometrically and refined as riding atoms with with C—H = 0.93 Å, and with *U*_eq_(H) = 1.2 *U*_eq_(C).

### Crystal data

**Table:**

[ZnCl_2_(C_11_H_9_N_3_)_2_]	*D*_x_ = 1.472 Mg m^−^^3^
*M**_r_* = 502.69	Mo *K*α radiation, λ = 0.71073 Å
Orthorhombic, *P**b**c**n*	Cell parameters from 9900 reflections
*a* = 13.7960 (4) Å	θ = 3.2–28.2°
*b* = 10.1905 (3) Å	µ = 1.34 mm^−^^1^
*c* = 16.1305 (5) Å	*T* = 298 K
*V* = 2267.76 (12) Å^3^	Block, orange
*Z* = 4	0.36 × 0.32 × 0.30 mm
*F*(000) = 1024	

### Data collection

**Table:**

Bruker APEXII CCD diffractometer	2160 reflections with *I* > 2σ(*I*)
φ and ω scans	*R*_int_ = 0.041
Absorption correction: multi-scan (*SADABS*; Bruker, 2013)	θ_max_ = 28.3°, θ_min_ = 3.5°
*T*_min_ = 0.708, *T*_max_ = 0.746	*h* = −18→18
65168 measured reflections	*k* = −13→13
2820 independent reflections	*l* = −21→21

### Refinement

**Table:**

Refinement on *F*^2^	0 restraints
Least-squares matrix: full	Hydrogen site location: inferred from neighbouring sites
*R*[*F*^2^ > 2σ(*F*^2^)] = 0.030	H-atom parameters constrained
*wR*(*F*^2^) = 0.083	*w* = 1/[σ^2^(*F*_o_^2^) + (0.0384*P*)^2^ + 0.7794*P*] where *P* = (*F*_o_^2^ + 2*F*_c_^2^)/3
*S* = 1.06	(Δ/σ)_max_ = 0.001
2820 reflections	Δρ_max_ = 0.35 e Å^−^^3^
141 parameters	Δρ_min_ = −0.22 e Å^−^^3^

### Special details

**Table:**

Geometry. All e.s.d.'s (except the e.s.d. in the dihedral angle between two l.s. planes) are estimated using the full covariance matrix. The cell e.s.d.'s are taken into account individually in the estimation of e.s.d.'s in distances, angles and torsion angles; correlations between e.s.d.'s in cell parameters are only used when they are defined by crystal symmetry. An approximate (isotropic) treatment of cell e.s.d.'s is used for estimating e.s.d.'s involving l.s. planes.

### Fractional atomic coordinates and isotropic or equivalent isotropic displacement parameters (Å^2^)

**Table:**

	*x*	*y*	*z*	*U*_iso_*/*U*_eq_	
Zn1	1.0000	0.21091 (3)	0.7500	0.04573 (11)	
Cl1	0.96785 (4)	0.08709 (5)	0.63805 (3)	0.06114 (15)	
N1	0.86977 (11)	0.30520 (15)	0.76959 (10)	0.0468 (3)	
N2	0.95560 (12)	0.45206 (15)	0.84789 (10)	0.0514 (4)	
N3	0.95301 (13)	0.55766 (16)	0.88646 (11)	0.0584 (4)	
C1	0.78854 (15)	0.2566 (2)	0.73676 (13)	0.0571 (5)	
H1	0.7927	0.1832	0.7026	0.069*	
C2	0.69884 (17)	0.3110 (2)	0.75155 (14)	0.0676 (6)	
H2	0.6434	0.2755	0.7275	0.081*	
C3	0.69288 (16)	0.4185 (2)	0.80247 (16)	0.0730 (6)	
H3	0.6330	0.4563	0.8140	0.088*	
C4	0.77541 (15)	0.4698 (2)	0.83614 (14)	0.0627 (5)	
H4	0.7726	0.5429	0.8706	0.075*	
C5	0.86318 (13)	0.41115 (18)	0.81815 (11)	0.0484 (4)	
C6	1.04406 (15)	0.60029 (19)	0.91730 (12)	0.0555 (5)	
C7	1.04401 (19)	0.7222 (2)	0.95484 (16)	0.0696 (6)	
H7	0.9866	0.7696	0.9593	0.084*	
C8	1.1291 (2)	0.7733 (3)	0.98561 (16)	0.0789 (7)	
H8	1.1293	0.8553	1.0110	0.095*	
C9	1.2132 (2)	0.7037 (3)	0.97888 (15)	0.0763 (7)	
H9	1.2707	0.7390	0.9990	0.092*	
C10	1.21338 (17)	0.5810 (2)	0.94239 (14)	0.0706 (6)	
H10	1.2709	0.5338	0.9386	0.085*	
C11	1.12915 (16)	0.5283 (2)	0.91168 (13)	0.0623 (5)	
H11	1.1291	0.4455	0.8874	0.075*	

### Atomic displacement parameters (Å^2^)

**Table:**

	*U*^11^	*U*^22^	*U*^33^	*U*^12^	*U*^13^	*U*^23^
Zn1	0.03382 (16)	0.04842 (18)	0.05494 (19)	0.000	0.00041 (11)	0.000
Cl1	0.0475 (3)	0.0725 (3)	0.0635 (3)	0.0004 (2)	−0.0018 (2)	−0.0153 (2)
N1	0.0390 (8)	0.0491 (8)	0.0522 (8)	0.0044 (6)	0.0020 (6)	0.0048 (6)
N2	0.0533 (9)	0.0493 (8)	0.0518 (9)	0.0059 (7)	0.0038 (7)	0.0004 (7)
N3	0.0597 (11)	0.0517 (9)	0.0638 (10)	0.0067 (8)	0.0061 (8)	−0.0026 (8)
C1	0.0431 (10)	0.0613 (11)	0.0669 (13)	0.0021 (9)	−0.0025 (8)	0.0013 (9)
C2	0.0393 (10)	0.0797 (15)	0.0837 (16)	0.0077 (10)	−0.0056 (10)	0.0097 (12)
C3	0.0490 (12)	0.0840 (16)	0.0861 (16)	0.0254 (11)	0.0070 (11)	0.0133 (13)
C4	0.0602 (12)	0.0615 (12)	0.0663 (12)	0.0211 (10)	0.0057 (10)	0.0027 (10)
C5	0.0472 (10)	0.0497 (10)	0.0483 (10)	0.0089 (8)	0.0034 (8)	0.0093 (8)
C6	0.0584 (12)	0.0551 (11)	0.0530 (11)	0.0001 (9)	0.0074 (9)	0.0013 (9)
C7	0.0696 (14)	0.0602 (13)	0.0790 (15)	0.0002 (10)	0.0151 (12)	−0.0122 (11)
C8	0.0851 (18)	0.0683 (15)	0.0832 (16)	−0.0170 (13)	0.0133 (14)	−0.0153 (12)
C9	0.0742 (16)	0.0856 (17)	0.0690 (14)	−0.0211 (13)	0.0029 (12)	0.0037 (12)
C10	0.0624 (13)	0.0789 (16)	0.0704 (14)	0.0030 (11)	0.0025 (11)	0.0065 (12)
C11	0.0657 (13)	0.0609 (12)	0.0602 (12)	0.0051 (10)	0.0035 (10)	−0.0020 (10)

### Geometric parameters (Å, º)

**Table:**

Zn1—N1	2.0618 (15)	C3—H3	0.9300
Zn1—N1^i^	2.0619 (15)	C4—C5	1.381 (3)
Zn1—Cl1^i^	2.2471 (5)	C4—H4	0.9300
Zn1—Cl1	2.2472 (5)	C6—C7	1.382 (3)
N1—C1	1.335 (3)	C6—C11	1.387 (3)
N1—C5	1.337 (2)	C7—C8	1.377 (3)
N2—N3	1.244 (2)	C7—H7	0.9300
N2—C5	1.425 (2)	C8—C9	1.364 (4)
N3—C6	1.419 (3)	C8—H8	0.9300
C1—C2	1.377 (3)	C9—C10	1.382 (3)
C1—H1	0.9300	C9—H9	0.9300
C2—C3	1.372 (3)	C10—C11	1.372 (3)
C2—H2	0.9300	C10—H10	0.9300
C3—C4	1.365 (3)	C11—H11	0.9300
			
N1—Zn1—N1^i^	124.45 (9)	C5—C4—H4	120.6
N1—Zn1—Cl1^i^	108.09 (4)	N1—C5—C4	122.15 (19)
N1^i^—Zn1—Cl1^i^	102.29 (5)	N1—C5—N2	111.89 (15)
N1—Zn1—Cl1	102.29 (5)	C4—C5—N2	125.96 (18)
N1^i^—Zn1—Cl1	108.09 (4)	C7—C6—C11	120.3 (2)
Cl1^i^—Zn1—Cl1	111.68 (3)	C7—C6—N3	115.36 (19)
C1—N1—C5	118.37 (17)	C11—C6—N3	124.36 (19)
C1—N1—Zn1	119.85 (13)	C8—C7—C6	119.8 (2)
C5—N1—Zn1	121.69 (13)	C8—C7—H7	120.1
N3—N2—C5	113.33 (16)	C6—C7—H7	120.1
N2—N3—C6	114.51 (17)	C9—C8—C7	120.0 (2)
N1—C1—C2	122.4 (2)	C9—C8—H8	120.0
N1—C1—H1	118.8	C7—C8—H8	120.0
C2—C1—H1	118.8	C8—C9—C10	120.4 (2)
C3—C2—C1	118.6 (2)	C8—C9—H9	119.8
C3—C2—H2	120.7	C10—C9—H9	119.8
C1—C2—H2	120.7	C11—C10—C9	120.4 (2)
C4—C3—C2	119.6 (2)	C11—C10—H10	119.8
C4—C3—H3	120.2	C9—C10—H10	119.8
C2—C3—H3	120.2	C10—C11—C6	119.1 (2)
C3—C4—C5	118.8 (2)	C10—C11—H11	120.5
C3—C4—H4	120.6	C6—C11—H11	120.5
			
C5—N2—N3—C6	179.59 (15)	N3—N2—C5—N1	173.32 (16)
C5—N1—C1—C2	−0.4 (3)	N3—N2—C5—C4	−7.2 (3)
Zn1—N1—C1—C2	176.25 (16)	N2—N3—C6—C7	175.28 (19)
N1—C1—C2—C3	−0.5 (3)	N2—N3—C6—C11	−4.7 (3)
C1—C2—C3—C4	0.9 (3)	C11—C6—C7—C8	1.0 (3)
C2—C3—C4—C5	−0.3 (3)	N3—C6—C7—C8	−179.0 (2)
C1—N1—C5—C4	1.0 (3)	C6—C7—C8—C9	0.1 (4)
Zn1—N1—C5—C4	−175.57 (14)	C7—C8—C9—C10	−1.0 (4)
C1—N1—C5—N2	−179.50 (16)	C8—C9—C10—C11	0.7 (4)
Zn1—N1—C5—N2	3.9 (2)	C9—C10—C11—C6	0.4 (3)
C3—C4—C5—N1	−0.7 (3)	C7—C6—C11—C10	−1.3 (3)
C3—C4—C5—N2	179.92 (19)	N3—C6—C11—C10	178.7 (2)

Symmetry code: (i) −*x*+2, *y*, −*z*+3/2.

### Hydrogen-bond geometry (Å, º)

**Table:**

*D*—H···*A*	*D*—H	H···*A*	*D*···*A*	*D*—H···*A*
C3—H3···Cl1^ii^	0.93	2.75	3.675 (2)	173
C1—H1···Cl1	0.93	2.81	3.411 (2)	124

Symmetry code: (ii) *x*−1/2, *y*+1/2, −*z*+3/2.
