# Supplementary figures and images for: Crystal structure of di­chlorido­bis­[2-(phenyl­diazen­yl)pyridine-κN 1]zinc
Source: Acta Crystallogr E Crystallogr Commun. 2015 Oct 24;71(Pt 11):m201–2. doi: 10.1107/S2056989015019143 (PMC4645076; doi:10.1107/S2056989015019143)

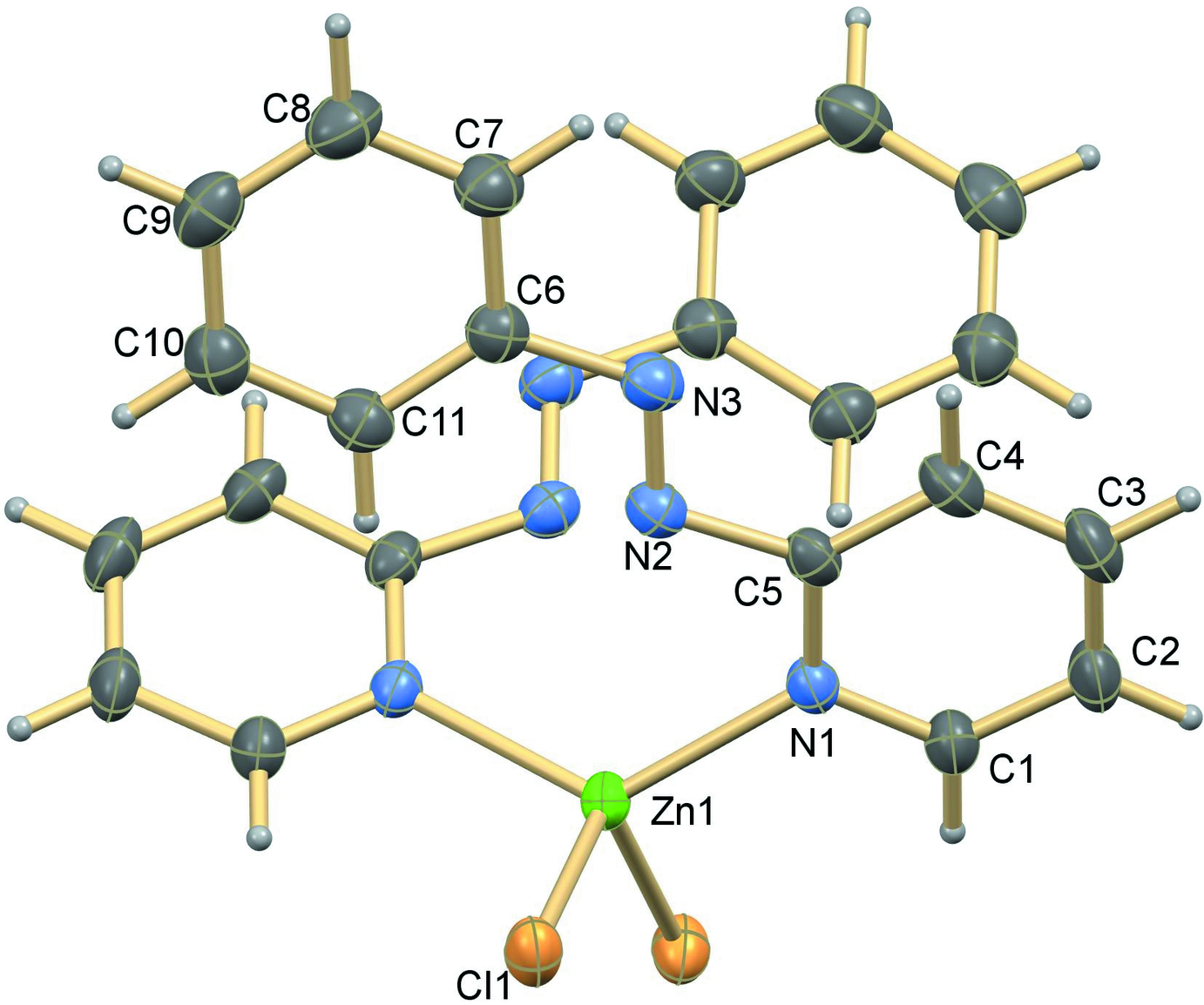

Supplement: Supplementary file 3 [file e-71-0m201-fig1.tif]

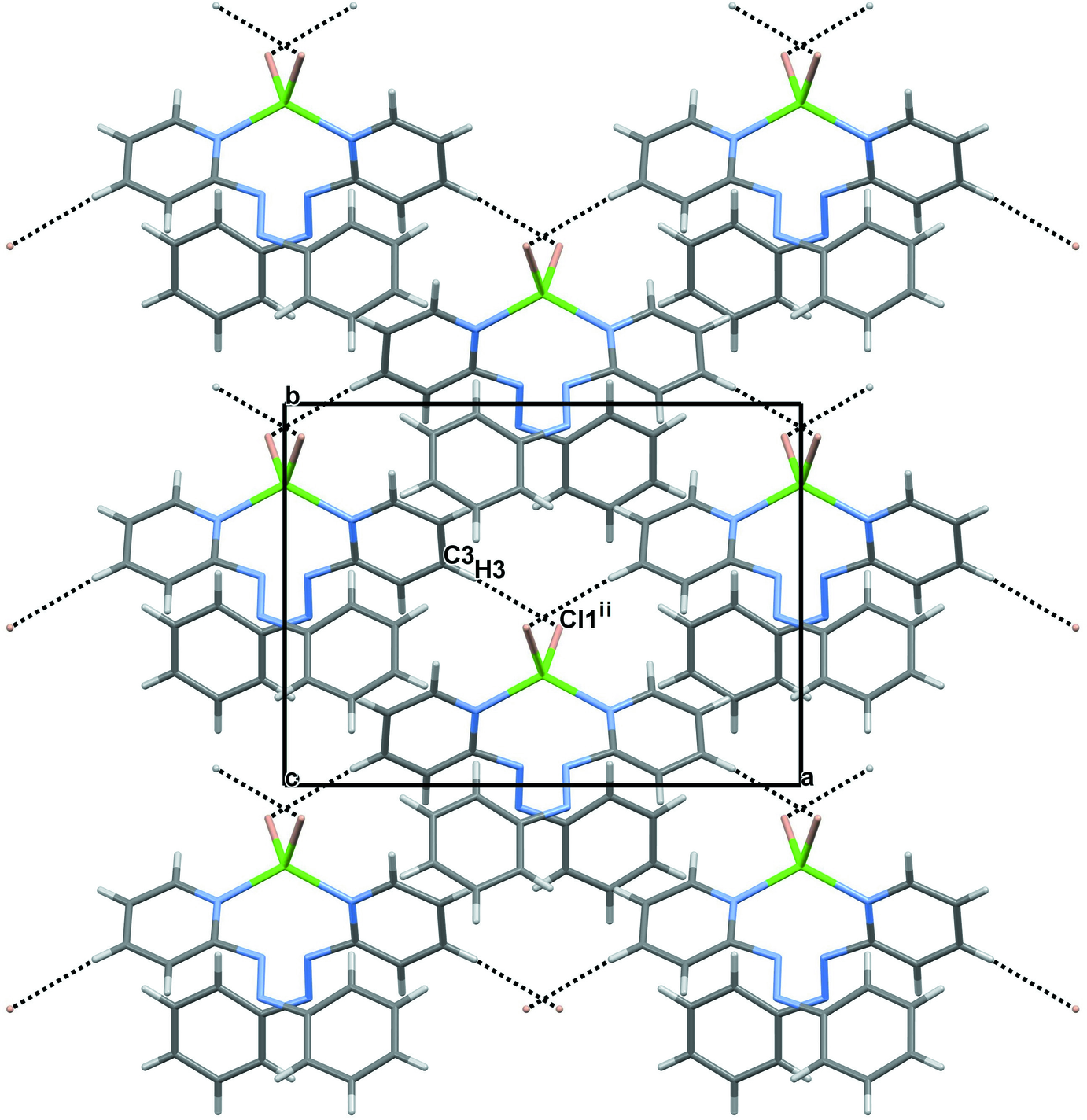

Supplement: Supplementary file 4 [file e-71-0m201-fig2.tif]

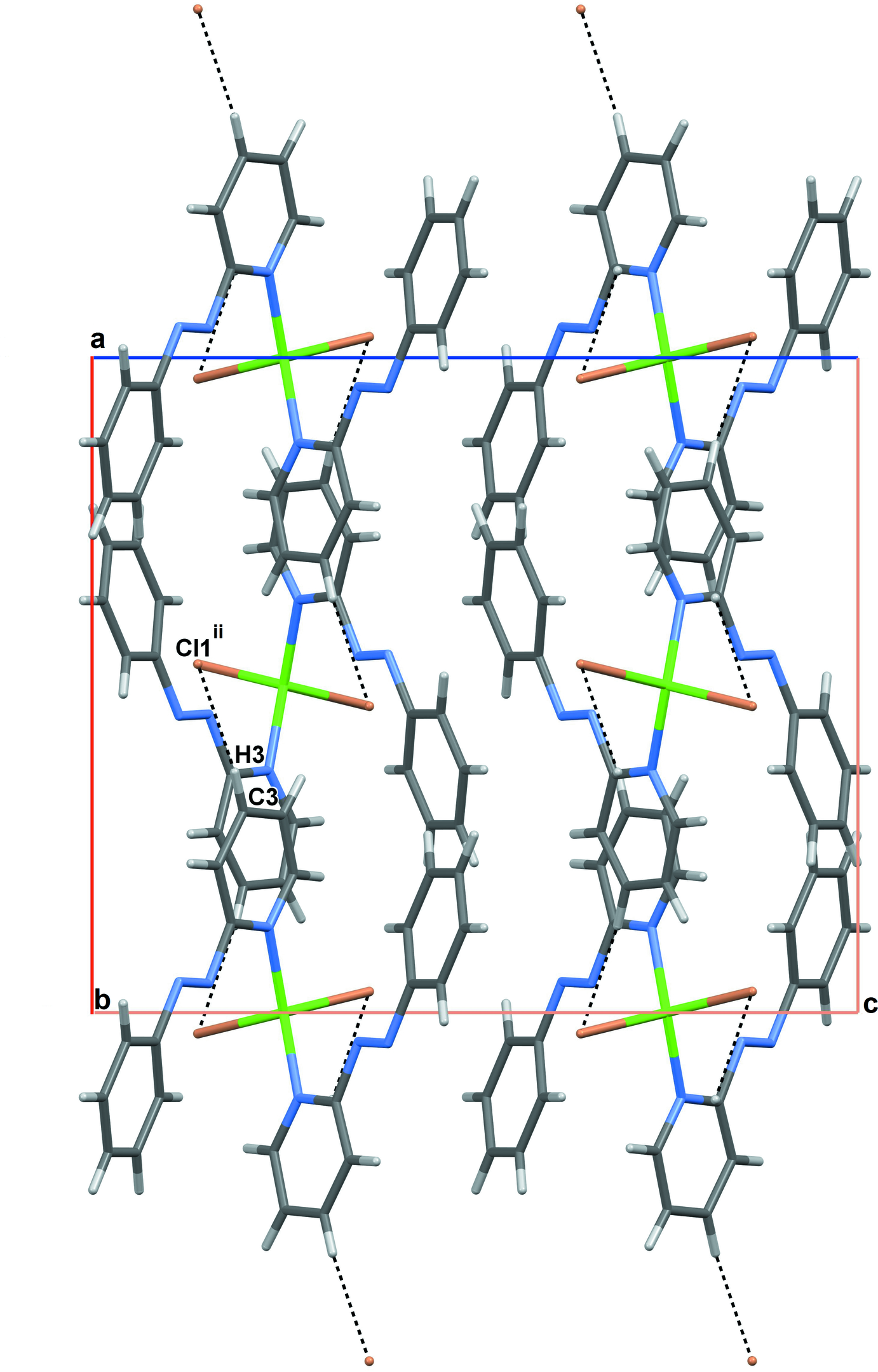

Supplement: Supplementary file 5 [file e-71-0m201-fig3.tif]

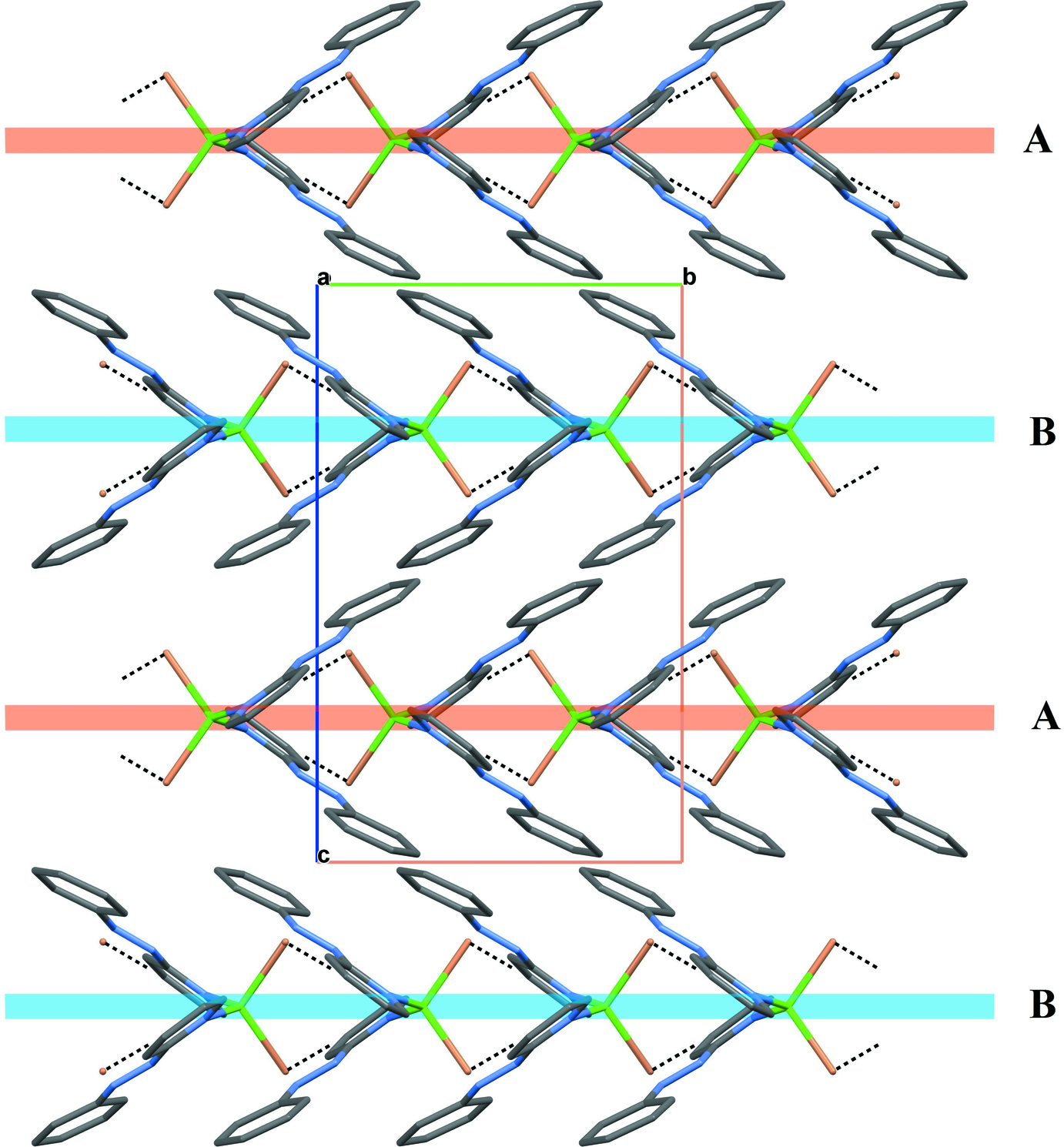

Supplement: Supplementary file 6 [file e-71-0m201-fig4.tif]
